# Genomic Characterization of Methicillin-Resistant *Staphylococcus aureus* (MRSA) by High-Throughput Sequencing in a Tertiary Care Hospital

**DOI:** 10.3390/genes11101219

**Published:** 2020-10-17

**Authors:** May Sherif Soliman, Noha Salah Soliman, Arwa Ramadan El-Manakhly, Shahira AbdelSalam ElBanna, Ramy Karam Aziz, Amani Ali El-Kholy

**Affiliations:** 1 Department of Clinical and Chemical Pathology, Faculty of Medicine, Cairo University, 11562 Cairo, Egypt; dr.may@maysherif.com (M.S.S.); nsal18@yahoo.com (N.S.S.); 2 Microbiology and Infection Control Department, Dar Al-Fouad Hospital, 11562 Cairo, Egypt; arwa.ramadan82@gmail.com; 3 Department of Microbiology and Immunology, Faculty of Pharmacy, Egyptian-Russian University, 11829 Badr City, Egypt; 4 Department of Microbiology and Immunology, Faculty of Pharmacy, Cairo University, 11562 Cairo, Egypt; shahira.ahmed@pharma.cu.edu.eg (S.A.E.); ramy.aziz@gmail.com (R.K.A.); 5 Center for Genome and Microbiome Research, Cairo University, 11562 Cairo, Egypt

**Keywords:** next-generation sequencing (NGS), whole-genome sequencing (WGS), methicillin- resistant *Staphylococcus aureus* (MRSA), virulome, resistome

## Abstract

Methicillin-resistant *Staphylococcus aureus* (MRSA) strains are associated with serious complications and poor clinical outcome. In Egypt, they contribute to more than 70% of *S. aureus* healthcare-associated infections. This study combined whole-genome sequencing, bioinformatics, and statistical analyses to identify the phylogeny, resistome, virulome and potential genotype–phenotype–clinical correlation among 18 clinical isolates of MRSA in a tertiary hospital in Cairo, Egypt. The ST1535-V MRSA clone was the most frequently isolated (16.6%), followed by ST5-VI, ST1-V and ST239-III (11.1% each). SCCmec V, VI, IV and III types were detected at frequencies of 50%, 16.6%, 11.1% and 11.1%, respectively. None of the tested virulence genes were detected in all isolates, but they ranged in distribution from 1/18 to 17/18. The Panton–Valentine leukocidin (PVL)-encoding genes were detected in only four isolates and were enriched in isolates causing non-severe cases. Phylogenetic analysis revealed relatedness between three ST1535-Vs, two ST5-VIs, two ST239-IIIs and two ST1-Vs; however, only the two genetically related ST1-V isolates were epidemiologically linked. While disease outcome and source of infection had no correlation with a particular genotypic pattern, the sequence type was the most correlated factor with phylogeny and genotypic patterns, and a few genes were associated with non-severe cases.

## 1. Introduction

*Staphylococcus aureus* is a bacterial pathogen that poses a serious public health threat and causes a wide range of infections. Methicillin-resistant *S. aureus* (MRSA) strains are particularly associated with serious complications and poor clinical outcome [1]. In one report, invasive infections by MRSA were estimated to be 94,360 in the United States, where they caused 18,650 deaths [2]. The length of hospital stay for MRSA reaches twice the length of other hospital stays, with much higher financial burden on hospitals [2].

In Egypt, MRSA strains constitute more than 70% of healthcare-associated *S. aureus* isolates and 11.5% of community-acquired *S. aureus* infections [3].

*S. aureus* is highly transmissible inside the hospital environment. Colonization and MRSA carriage by patients and healthcare workers act as sources of infections [4]. The evidence of outbreaks or transmission events can be investigated by various typing methods to study the relatedness of strains and track the source of infection [4]. Traditional methods of bacterial typing, such as antibiograms, PFGE and multi-locus sequence typing (MLST), are limited by their low resolution power and inability to discriminate closely related bacterial strains [2].

Evolution of *S. aureus* occurs through single-nucleotide variants (SNVs) and point mutations at a high rate, estimated to be 3.4 × 10^−6^ mutations per site per year, with approximately one different SNV every 5–10 weeks [4]. Accordingly, MRSA isolates that belong to a single lineage cannot be discriminated by the usual typing techniques [2]. This limitation has been overcome by the rise of whole-genome sequencing (WGS) [4], which offers high-resolution analysis of pathogens’ microevolution [5]. High-throughput sequencing (HTS, popularly known as next-generation sequencing or NGS) is used for WGS to provide a full description of a bacterial genome and offer all genetic sequence data required to reach the highest level of microbial identification, and to predict virulence and antimicrobial resistance [1,4].

The aim of this study was to investigate the clonality of clinical isolates of MRSA in a tertiary hospital in Cairo, Egypt, identify their patterns of resistance and virulence genes and investigate correlations between these genotypic patterns and clinical, epidemiological and phenotypic characteristics.

## 2. Materials and Methods

### 2.1. Ethical Statement

All study protocols were approved by the Institutional Review Board of the Faculty of Medicine, Cairo University (IRB Approval: 07072018).

### 2.2. Bacterial Identification and Drug Susceptibility Testing

This study included 18 *S. aureus* isolates from non-duplicated samples of patients with variable types of infections, who were admitted in wards and intensive care units (ICUs) of a major tertiary care hospital in Egypt between 2017 and 2018 (Table 1 and Table S1). All the samples were delivered to the microbiology laboratory with optimal transport conditions for routine workup. *S. aureus* was isolated on blood and chocolate agar media (Oxoid, Basingstoke, UK) after incubation at 36 °C (±1 °C) for 16–18 h and was identified by standard microbiological procedures [6]. The antimicrobial susceptibility of each isolate was phenotypically tested by the standard Kirby Bauer disc diffusion method, according to the CLSI guidelines [7]. As disc diffusion is not reliable for testing susceptibility to vancomycin, the minimal inhibitory concentration was determined by E-test strips (BioMérieux, Lyon, France). Quality control measures were assured by the use of *S. aureus* ATCC 25923 as a standard strain. All isolates were stored in trypticase soy broth, supplied with 10% glycerol at −70 °C for further analysis.

### 2.3. High-Throughput Sequencing (HTS) on Illumina MiSeq Platform

Bacterial DNA was extracted from freshly subcultured colonies by the QIAamp DNA Mini Kit (Qiagen, Valencia, CA, USA, cat # 51304), according to the manufacturer’s instructions, after preliminary incubation with lysozyme at 37 °C for 1 h. The DNA concentration was accurately measured in a DeNovix fluorometer (DeNovix Inc., USA). The genomic DNA was stored at −20 °C. One nanogram of bacterial DNA was used in the library preparation. The library was prepared with Nextera XT DNA Library Preparation Kit (FC-131-1096, Illumina, La Jolla, CA, USA), according to the manufacturer’s instructions. In brief, transposons were used to fragment DNA, then adapter sequences were added to the DNA template. The product was then enriched and quantified by the DeNovix dsDNA High Sensitivity Assay (DeNovix Inc., Wilmington, DE, USA). Bead-based normalization in Nextera-XT DNA Library Preparation Kits was used. Equal volumes of each normalized library were pooled in a single tube, diluted, then heat-denatured, according to the MiSeq System Denature and Dilute Libraries Guide (Protocol B). The library products were sequenced in an Illumina MiSeq instrument, supplied with a reagent kit V3 600 cycles (Illumina, La Jolla, CA, USA).

### 2.4. Bioinformatics Analysis

Raw reads were pre-processed by the Illumina in-house software for adapter removal and preliminary trimming. Seqtk (https://github.com/lh3/seqtk/) was further used for trimming low-quality sequences and short reads. FastQC [8] was used for quality check after each of the pre-processing steps. SPAdes [9] (version 3.11.1) was used for *de novo* assembly of the sequences, which were also validated with the built-in SPAdes assembly service offered by the PATRIC resource center (https://patricbrc.org). Seqtk was used once more to remove contigs smaller than 200 bp, after assembly and prior to annotation. Finally, all trimmed and cleaned contigs were annotated in PATRIC. The Center of Genomic Epidemiology Website services were used in the annotation of the assembled sequences, as follows: ResFinder 3.0 and VirulenceFinder 2.0 were used for identification of different resistance and virulence genes, respectively, whereas spaTyper 1.0 and SCCmecFinder were used for Spa and SCCmec typing, respectively [10,11,12].

The phylogenetic tree was built by the tool “Codon Trees,” which could be seen as a phylogenomic analysis tool provided by PATRIC [13]. Codon Trees uses a defined number of randomly picked conserved global protein families (PGFams) [14] (here, 1000 families were used), to build an alignment. The phylogenetic tree is generated on the basis of differences within those selected sequences. Protein sequences are aligned with MUSCLE [15], and nucleotide sequences are aligned by the Codon_align function of BioPython [16]. Finally, RaxML [17] was used to compute distances and final trees. Support values are generated by 100 rounds of the rapid bootstrapping option [18] in RaxML. FigTree (http://tree.bio.ed.ac.uk/software/figtree/) was used for the graphical representation of the tree.

### 2.5. Statistical Analysis

Relations between categorical data were analyzed by contingency tables, and statistical significance was assessed by Fisher’s exact test, with Monte Carlo simulations for contingency tables with >2 × 2 cells. Associations between binary variables (presence/absence) were analyzed by Pearson correlation, in addition to Fisher’s exact test. Hierarchical clustering analysis was performed by the default algorithm of the R *heatmap* function. GraphPad Prism (GraphPad Software, San Diego, CA, USA) was used for standard statistical tests, and the R statistical platform (https://www.r-project.org) and RStudio program (https://rstudio.com/) were used for large-scale analysis and data visualization. R packages used included *readxl* and *corrplot*.

### 2.6. Data Accessibility

All genome sequences were deposited in NCBI, as a part of the bioproject PRJNA658679 and were assigned unique accession numbers (Table 2).

## 3. Results

### 3.1. Nature and Source of the Isolates

This study included 18 MRSA isolates that had been confirmed to harbor the SCC*mecA* gene, known for encoding methicillin resistance (Table 1). They were recovered from different clinical specimens, mainly blood (6/18) and endotracheal aspirate (ETA, 5/18) as well as tissue, wound and prosthetic devices (Table 1, Supplementary Table S1).

Sequencing the genomes of these isolates showed that they belong to different MRSA clones with different multi-locus sequence types (ST) and SCC*mec* cassettes (Table 1). Three isolates (16.6%) belonged to ST1535-V MRSA, while ST5-VI, ST1-V and ST239-III were each represented by two isolates (11.1%) and two MRSA isolates had an undetermined sequence type (STu). MRSA isolates mostly harbored SCC*mec*-V, -VI, -IV and -III types with frequencies of 50%, 16.6%, 11.1% and 11.1%, respectively. One MRSA isolate (ST8-like) had an untypeable SCC*mec* element (Table 1, Supplementary Table S1).

Intensive care units (ICUs) were the source of 33.3% (6/18) of MRSA isolates with clones found to be ST8-V, ST8-like, ST5-VI, ST239-III and ST22-IV. Nine healthcare-associated (HA-MRSA) isolates (50%) were acquired by patients after more than 48 h of hospital admission, including five isolates from healthcare-associated infections and four isolates from healthcare-acquired colonization. The identified clones of HA-MRSA isolates were found to be ST8-V, ST1-like-V, ST1535-V, ST22-IV, ST8-like, ST80-IV, ST5-VI and ST121-V.

With the relatively small number of isolates analyzed, no statistically significant association was found between STs and the specimen or source of isolates (type of hospital ward or whether it is community-acquired or hospital-acquired).

### 3.2. Virulome Analysis

Genome sequencing allowed the reconstruction of the virulomes of the isolates, i.e., the comprehensive detection and classification of virulence genes they carry (Table 3). At least one gene belonging to the groups of hemolysins and serine proteases was detected in 94.4% and 88.8% of MRSA isolates, respectively (Table 3). Genes encoding aureolysins, leukocidins, *Staphylococcus* complement inhibitor (SCIN), enterotoxins and staphylokinases were detected at frequencies of 88.8%, 88.8%, 72.2%, 66.6% and 61.1% of all MRSA isolates, respectively. The Panton–Valentine leukocidin (PVL)-encoding genes were only present in four isolates (22.2%), of which three were recovered from wounds and belonged to ST1-like-V, ST8-V and ST1-V clones, while one PVL-positive isolate belonged to the ST121-V clone and was recovered from an infected prosthesis. The absence of the PVL-encoding genes, *lukS* and *lukF*, in bacteria isolated from blood and ETA was remarkable and statistically significant (Fisher’s exact test *p*-value = 0.005), as is further detailed in the last section of the Results section.

Each of the toxic shock syndrome toxin (TSST) and exfoliative toxin (*eta*) genes was only detected in one isolate. The TSST-encoding gene (*tst*) was present in the genome of an ST22-IV MRSA isolate recovered from an ETA sample from a patient, admitted to a medical ICU, who passed away, while *eta* was detected in an ST913-V MRSA isolate from a tissue sample of a patient in a surgical ward.

### 3.3. Resistome Analysis

Given that these isolates were selected for being methicillin-resistant, the determination of the complete set of known resistance genes, i.e., the resistome, was necessary to define the full resistance potential of the isolates, and its implication on their potential to evolve into pan-resistant ones.

Expectedly, co-occurrence of the β-lactam resistance genes, *mecA* and *blaZ*, was confirmed in all isolates. Resistance genes to fluoroquinolone, aminoglycoside, tetracycline, macrolides, lincosamide and trimethoprim-sulfamethoxazole were detected at frequencies of 94.4%, 72.2%, 61.1%, 27.7%, 16.6% and 5.5%, respectively (Table 4, Supplementary Table S1). One of the two isolates from undetermined sequence types (STu) carried *mphC*, a gene that encodes macrolide-lincosamide-streptogramin (MLS) resistance. A fusidic acid resistance-conferring gene was detected once.

The presence of resistance genes within these 18 genomes was not necessarily in accordance with resistance phenotypes, which were determined for representative antibiotic classes (Table 4).

### 3.4. Phylogeny and Genetic Relatedness of the Sequenced Isolates:

**Figure 1:**
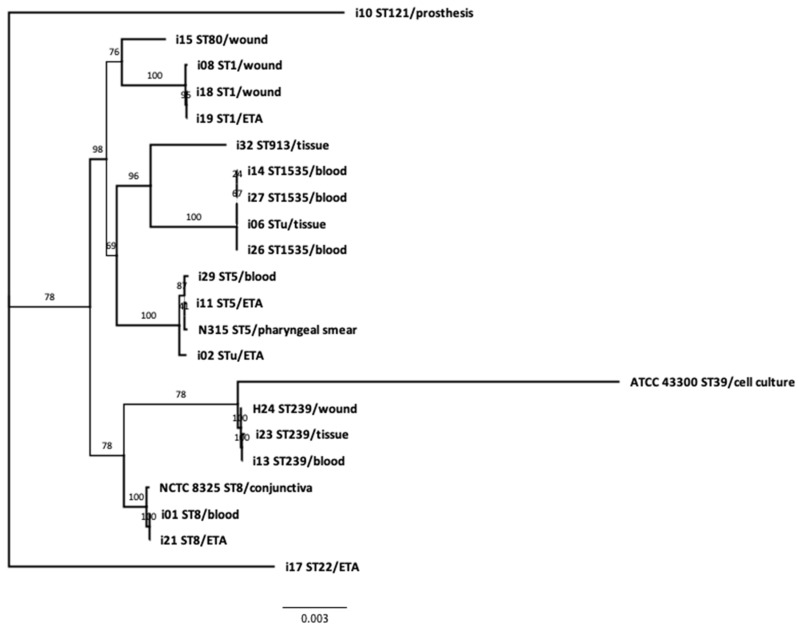
Codon tree-based phylogenetic analysis of MRSA isolates showing genetically linked MRSA clones. Isolate codes and sequence types, as well as source of isolation, are shown. *S. aureus* ATCC 43300, sequenced as control in this work, as well as NCTC 8325 (ST8) and N315 (ST5), were used as reference isolates. *S. aureus* H24, being the only fully sequenced isolate with confirmed geographical origin in Egypt (see Discussion), is used for comparison. The phylogenetic tree was built with the PATRIC pipeline, with a set of 1000 conserved core genes.

Phylogenetic analysis confirmed the genetic relatedness of MRSA isolates that have the same ST types and SCC*mec* cassettes (Figure 1). The analysis highlighted the relatedness of ST1-like isolate 8 to ST1 isolates 18 and 19, and ST8-like isolate 21 to ST8 isolate 1. Isolate 2, with unknown ST, was in close relatedness to ST5 isolates 11 and 29, while isolate 6 was closely related to ST1535 isolates 14, 26 and 27.

**Figure 2:**
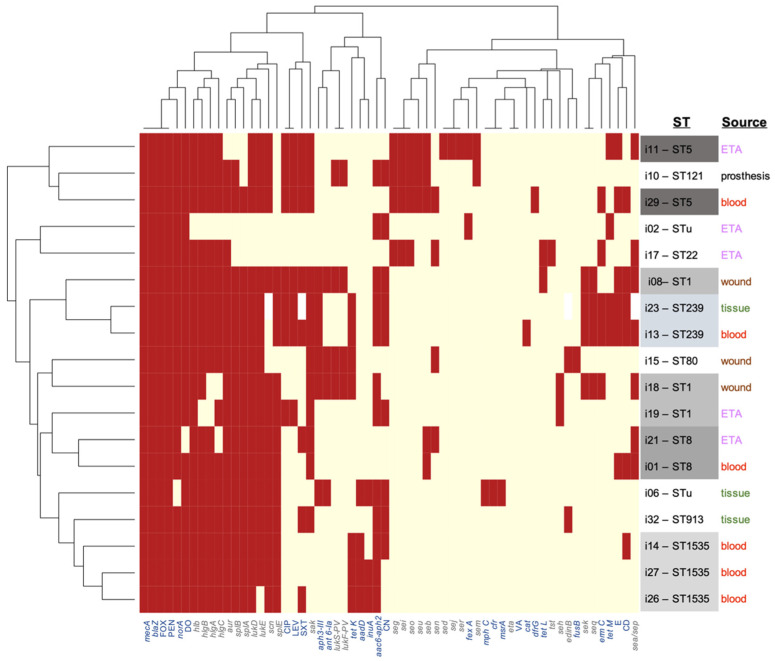
Hierarchical clustering of the isolates (vertical axis) against the combined pattern of resistance and virulence genotypes as well as the antibiotic susceptibility phenotypes. The figure highlights the resistome and virulome genetic profiles as well as the unsupervised clustering of isolates which parallels their sequence type classification. Resistance genes or phenotypes are labeled in blue (X axis), with italics and straight letters, respectively. Virulence genes are labeled in gray. Similar sequence types are highlighted with similar shades of gray next to isolate numbers. Specimen origin is also color-coded by source (right annotations). The figure was generated in R by the heatmap package using default clustering settings. Brick color = presence of gene (or resistant phenotype). Pale yellow = gene absence (or susceptibility phenotype).

A hierarchically clustered colored matrix (Figure 2) shows correspondent virulence and resistance profiles between the genetically related MRSA strains. Intriguingly, this clustering—solely based on virulome and resistome profiles—is in strong concordance with the phylogeny and the sequence typing, emphasizing two major findings of this work: (i) the lack of clonality among these isolates from the same hospital and (ii) the ability of isolates from multiple genetic backgrounds and multiple assortments of virulence genes to cause similar clinical manifestations and outcomes.

Only two genetically related isolates of the ST1-V MRSA clone, isolates 18 and 19, were recovered from two patients residing in the same ward (MSU6) with a two-month interval (Table 1). Otherwise, the rest of the genetically linked isolates were recovered from different hospital wards at distant periods.

Of note, the patients who died (5/18) had isolates from various STs, three of which were from blood samples and belonged to ST8-V, ST1535-V and ST239-III, while the other two were ETA samples, and belonged to ST8-like and ST22-IV. All of these patients, except the one who had ST1535-V MRSA in blood, were in ICUs. Not surprisingly, ICU stay was associated with death (Fisher’s test *p*-value = 0.0217; odds ratio = 22.0).

### 3.5. Clinicoepidemiological–Genotype Correlations

Like with several WGS studies, the purpose of this study was not primarily epidemiological, as the in-depth analysis offered by HTS is traded off for the robust statistics offered by the large number of specimens analyzed with other less expensive typing/screening methods. In spite of the limited number of isolates, which does not grant statistical power, comparing the clinicoepidemiolgical parameters available (e.g., clinical specimen, diagnosis, hospital ward, source of acquisition, severity of disease) to virulome and resistome patterns still delineated some interesting associations (Supplementary Table S2, Supplementary Data Sheet 1).

First, a few associations among the available clinical and epidemiological parameters were deduced: for example, an evident statistical dependence was observed between sample types and diagnosis (Fisher’s exact test *p*-value = 0.002, Supplementary Table S2). Obviously, severity was directly determined by the specimen type (severe cases were those in which blood or ETA was septic, while non-severe ones had bacteria isolated from tissues or peripheral organs).

In terms of relations with gene presence and absence patterns, as well as resistance phenotypes, no relation was found between the source of acquisition (community- or hospital-acquired or colonization-associated) and any particular genetic pattern. Similarly, and intriguingly, there was no association between clinical outcome (death or discharge) and any genetic or virulence pattern.

Meanwhile, a few intriguing associations were uncovered between severity and some virulence or resistance genes: for example, no PVL leukocidin gene was found in any severe cases (Fisher’s test *p*-value = 0.011), and this observation was true for sample type (blood, tissue and ETA isolates were all PVL-negative, while all three wound isolates had the genes, as stated above). Likewise, two aminoglycoside resistance genes (*ant 6-Ia* and *aph(3′)-III*) were reverse-associated with case severity. The gene *ant 6-Ia* was absent in all isolates from severe cases (Fisher’s test *p*-value = 0.011) and particularly associated with wound infections (Fisher’s test *p*-value = 0.005), while *aph (3′)-III* was absent in all but one isolate from severe cases (Fisher’s test *p*-value = 0.013). On the other hand, no particular genes were positively associated with case severity.

### 3.6. Phenotype-Genotype Correlations

The question of phenotype–genotype and gene–gene correlations is a more complex one, given the relatively large number of resistance and virulence genes that could be compared. Obvious correlations exist between chromosomal neighbors such as genes on one operon or co-localized genes on genomic islands (e.g., *lukS.PV* and *lukF.PV*)

**Figure 3:**
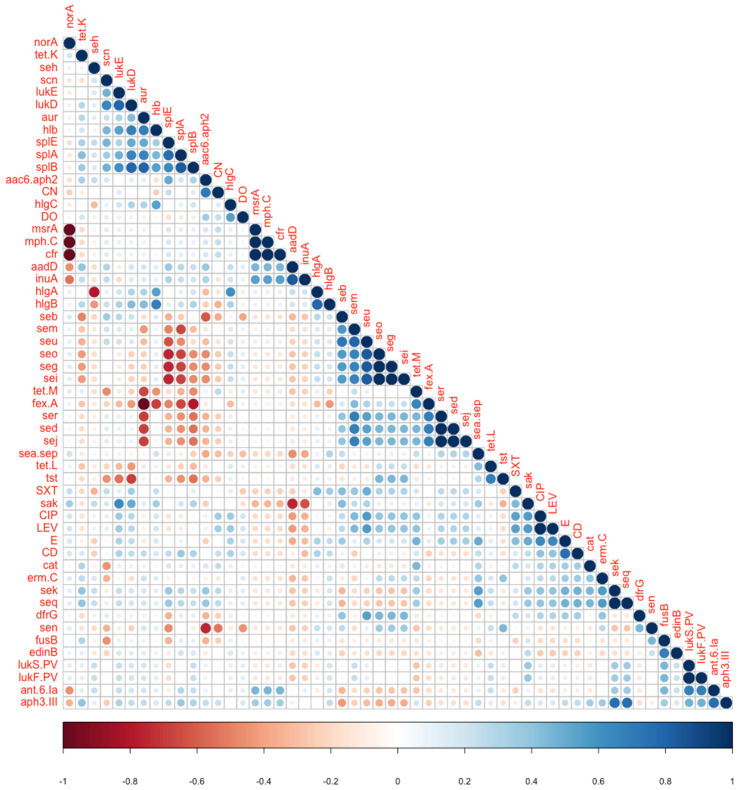
Correlation matrix indicating all correlations between pairs of genetic or phenotypic determinants (resistance phenotypes). Shades of blue represent positive correlations, while shades of orange/red represent negative correlations. Similar analyses were conducted separately among resistance-related pairs of traits and virulence-related ones (Supplementary Figure S1).

To systematically detect all such associations in an unbiased way, we analyzed all potential pairs of variables (except those which were entirely present or completely absent among the 18 isolates, e.g., *mec* and *blaZ*) in contingency tables, and statistically assessed their dependence by Fisher’s exact test (which is the test of choice for those small frequencies, Supplementary Table S2). In addition, using a pseudo-numeric binary matrix (in which presence or resistance is marked as 1 and absence or susceptibility as 0, Supplementary Table S1), we estimated the correlation coefficients among all potential pairs (Figure 3), as well as among resistance-related pairs and virulence-related pairs separately (Supplementary Figure S1).

Among the observed correlations are negative ones between the near ubiquitous quinolone resistance gene, *norA*, and some other rarely present resistance genes, e.g., *msrA* and *cfr*; and between some of the enterotoxin genes, e.g., *seg* and *seo*, and the serine protease genes, *splA* and *splE* (Table 5, Supplementary Table S2).

Other positive correlations were observed, in general, among different enterotoxin genes, or between the aureolysin gene, *aur*, and *splB* (Fisher’s test *p*-value = 0.0196). *lukS.PV* and *lukF.PV* were positively correlated with *ant.6.Ia* (Fisher’s test *p*-value = 0.019), while *lukD* was positively correlated with *splA* and *splB* (Fisher’s test *p*-value = 0.0392 and 0.0196, respectively).

## 4. Discussion

The past decade has witnessed a massive expansion in WGS of bacterial isolates, especially those from antimicrobial-resistant pathogenic microorganisms with public health relevance. While thousands of *S. aureus* genome sequences are publicly available, these sequences are disproportionately coming from a small number of countries, and thus do not represent the actual global diversity of this common pathogen. For example, the PATRIC database [13] lists 14,566 *S. aureus* genomes in its version 3.6.5 (accessed 9 August 2020). Meanwhile, 5065 of these genomes come from USA isolates, 561 from China, 545 from the UK and 508 from the Netherlands, and only one is reportedly from an Egyptian isolate (*Staphylococcus aureus* strain H24, PATRIC ID: 1280.4183) [19]. A more comprehensive picture of the distribution and lineages of *S. aureus* (notably MRSA) would be reached if a global representation of all isolates is achieved. To this end, we aimed, in this study, to genomically characterize 18 MRSA isolates, from an Egyptian hospital, and study their clonal relatedness using the WGS technology.

The molecular epidemiology of MRSA is variable among different geographical areas, and each healthcare setting has its unique pattern [20,21]. The most commonly detected MRSA clones in our study were ST1535 (16.6%), ST239 (11.1%), ST5 (11.1%) and ST1 (11.1%). In the present study, two isolates had an undetermined sequence type (STu). According to other studies, an undetermined sequence type could be explained by fragmentation of the genome that affects marker calling [22]. The determination of sequence type is easily affected by genome fragmentation, without any effect on phylogeny, as the fragmentation involves few markers among 2213 markers, which is one more advantage for WGS [22].

In the present study, the most frequently detected SCC*mec* types harbored by MRSA isolates were SCC*mec*-V (50%), followed by SCC*mec*-VI (16.6%), then SCC*mec*-IV (11.1%) and SCC*mec*-III (11.1%). This is in accordance with previous studies in Egypt, which reported that most MRSA isolates harbored SCC*mec*-V and SCC*mec*-IV [23] and in line with several reports in the Middle East and Gulf area [20,24]. Few of those reports differed from our study in that SCC*mec*-IV was dominant over the SCC*mec*-V type. In another study from Egypt, SCC*mec*-I,-II and -III elements dominated [25]. This is an example of how each healthcare setting has its unique pattern of MRSA clones and its local epidemiology. For example, in Asian countries, SCC*mec*-III-containing MRSA clones tend to dominate [21], whereas in European and American countries, SCC*mec*-IV MRSA clones are the most predominant [26,27]. In line with our results, several studies agreed that SCC*mec*-I,-II and -III elements are the most predominant among HA-MRSA, while SCC*mec*-IV and -V were the most reported among CA-MRSA [21]. We identified one MRSA isolate with an untypeable SCC*mec* element which could be due to either *ccr* gene deletion or *ccr*-independent transfer of the *mecA* gene [25].

Regarding the virulome of the isolates, we identified the co-existence of multiple virulence groups. The genes of hemolysins and serine proteases were the most frequently detected (94.4% and 88.8%, respectively), followed by aureolysins and leukocidins (88.8%), SCIN (72.2%) and enterotoxins (66.6%). Our results confirm the findings of a previous study in Egypt, in which leukocidin and hemolysin genes existed with rates of 89.7% and 76.6%, respectively [28]. Similar frequencies were reported in studies from other countries [29].

PVL is a strong exotoxin that is more commonly reported in CA-MRSA strains [1]. In the present study, *Luk-PV* genes were detected in four isolates (ST1-V, ST1-like-V, ST80-IV and ST121-V MRSA) with a frequency of 22.2%, which agrees with previous studies in Gaza (29.5%) and Egypt (19%) [24,30]. Among the four PVL-positive MRSA, two isolates were HA-MRSA. Some studies reported a high prevalence of PVL among HA-MRSA, which may indicate that PVL-positive MRSA started to invade hospitals [20]. Although PVL genes have been usually recognized as a key marker for CA-MRSA, the horizontal gene transfer of PVL genes across variants of MRSA clones resulted into blurred distinctive boundaries between CA- and HA-MRSA strains. For example, a study in Egypt and Saudi Arabia reported an unusual low prevalence of PVL among the CA-MRSA isolated from nasal carriers [23]. Of more interest, the PVL-encoding genes detected in this work were all from non-severe cases, and no blood, tissue or ETA specimens had isolates that were PVL-positive. Association of PVL with non-severe cases has been recognized [31,32], but the reverse was also sporadically reported [33].

In our study, each of the TSST (*tst*) and exfoliative toxin (*eta*) genes was detected once in ST22-IV and ST913-V MRSA clones isolated from ETA and tissue samples of patients with chest infection and diabetic foot, respectively. Likewise, a previous study in Egypt reported the *tst* gene in 4.5% of isolates [28]; however, higher frequencies were reported in other geographical areas, such as Gaza [24].

In our study, all MRSA isolates showed coexistence of *mecA* and *blaZ* genes encoding PBP2a with low β-lactam affinity and a β-lactamase, respectively. All isolates lacked the vancomycin resistance gene. Resistance genes were mostly detected for ciprofloxacin, levofloxacin (94.4%), gentamycin (72.2%), doxycycline (61.1%) and erythromycin (33.5%). This is in line with previous reports from Egypt and other countries [1,25,28,34].

In the present study, most MRSA isolates (76.1%) showed phenotypic and genotypic concordance in antibiotic susceptibility results. Similar findings were reported in other studies that showed concordance in 87–100% of isolates [1,34]. Discordant results were recorded in 23.8% of isolates and were all among the non-β lactam antibiotics. Discordant results could be either due to isolates having an unexpressed resistance gene or isolates lacking the resistance gene, but conferring other mechanisms of resistance.

In the literature, most of the described members of *S. aureus* clonal complex CC-15 were methicillin-sensitive (MSSA). CC-15 MRSA was sporadically reported with limited genomic data. In the Gulf area, the first reported CC-15 MRSA ST1535 isolates were in Iran and Saudi Arabia and were associated with nasal colonization [35]. Interestingly, in the present study, ST1535 was the most frequent MRSA clone in our hospital, which is considered of high concern. Three ST1535-V MRSA isolates were recovered from blood samples of three patients. These three isolates had a nearly identical toxin profile and resistome, which was similar to ST1535 MRSA isolates reported in Saudi Arabia. [35].

In the present study, the isolation of two ST239-III MRSA isolates is another finding of interest, as this strain is considered the oldest MRSA clone that has caused pandemics and is still widespread worldwide [36]. Several reports describe the isolation of ST239-III MRSA from diverse geographical countries, including Egypt [36,37].

Other detected STs were ST22-IV, ST80-IV and ST8-V MRSA clones, which are of concern owing to their evolutionary and epidemiological history. ST22-IV MRSA was detected once from an ETA sample of an ICU patient with fatal outcome. This clone has the same ST as the UK-EMRSA-15, a well-known dominant European clone that appeared in the UK and established itself in Europe [38]. In our study, this strain was susceptible to erythromycin and ciprofloxacin, although it carried *ermC* and *norA*, *a* common feature of EMRSA-15 [38]. The ST22 isolate in our study was PVL-negative and *tst*-positive, which was similarly reported [20].

ST80-IV is another predominant MRSA clone in Europe and was shown by phylogenetic analysis to have African origins [39]. This clone has been frequently reported in several countries of the Middle East including Egypt [37,38,39]. The ST80-IV MRSA isolate in our study was PVL-positive, a common feature of this clone [20].

ST8-V MRSA was isolated once in our study from blood of a patient who died later. Apart from being PVL-negative, this strain is close to the ST8-IV USA300 PVL-positive, multidrug-resistant MRSA clone that has been identified as a cause of MRSA infections in North America, and has lately been increasingly reported worldwide [27,38].

The phylogenetic analysis of our isolates demonstrated close genetic relatedness between three ST1535-V, two ST5-VI, two ST239-III and two ST1-V MRSA isolates. The ST1-like isolate was shown to be genetically related to the ST1-V MRSA isolate, and the same was true for the ST8-like isolate that was found to belong to the ST8-V MRSA clone. A finding of interest in this study was that the ST was quite associated with genetic patterns and phylogeny, in spite of the low number of samples (Figure 1 and Figure 2). Consequently, the identity of STu isolates could be deduced from their phylogenetically related neighbors.

In the present study, two genetically related ST1-V MRSAs were isolated from two patients in the same ward within a period of two months, suggesting a transmission event. Otherwise, the rest of the genetically linked MRSA isolates were recovered from diverse clinical sources and different wards at wide gap periods and were mostly community-acquired, which excludes in-hospital transmission.

Finally, although the primary goal of the study was not to focus on the association of certain genes with the clinical outcome or case severity, all possible associations between variables were explored (Supplementary Table S1, Figure 2 and Figure 3 and Supplementary Figure S1). Of interest, unlike the strong clustering between STs and phylogenetic distance as well as genotypic patterns, there were no consistent associations between genotypes and isolate sources (hospital vs. community) or clinical outcome (death or discharge). This can always be attributed to the small number of isolates, but also to the fact that death is rarely a direct consequence of the infection or the causative isolate, but is often a function of the human immune/inflammatory response, other environmental factors and underlying health conditions. Still, the few associations that were statistically and biologically significant were rather with non-severe cases (e.g., PVL leukocidin genes and some other resistance genes were enriched in isolates from non-severe infections (Table 5)). Similar types of analyses observed similar patterns, e.g., with the *norA* resistance gene [40].

In conclusion, the use of HTS in the present study added value to the classic isolate identification and typing protocols, and offered precious genomic data about MRSA clones in our hospital, the most prevalent of which was ST1535-V. Multiple virulence and resistance genes were identified. No clonal relatedness was detected except for two genetically related ST1-V isolates recovered from the same hospital ward. Wide-scale studies are recommended to develop more knowledge about the genetic diversity of MRSA clones and their evolution inside hospitals.

## Figures and Tables

**Table 1:** Clinical, epidemiological and molecular characteristics of methicillin-resistant *S. aureus* (MRSA) isolates (*n* = 18).

Sequence Types	SCCmec Element Type * (*ccr* Complex Type)	Count and Frequency (%)	Isolate No. (Date of Isolation)	Diagnosis	Sample	Ward/Unit	Acquisition	Outcome
ST8	V(5C2)	1 (5.5%)	i01 (3/6/2018)	Sepsis	Blood	SICU2	HA	Death
STu	I(1B), VI(4B)	2 (11.1%)	i02 (5/5/2018)	CAUTI	ETA	MICU	CO	DC
STu	VI(4b)		i06 (11/4/2018)	SSI	Tissue	MSU1	CA	DC
ST1-like	V(5C2 and 5)	1 (5.5%)	i08 (6/4/2018)	Osteomyelitis	Wound	MSU5	CA	DC
ST121	V(5C2)	1 (5.5%)	i10 (3/4/2018)	Infected prosthesis	Infected prothesis	MSU6	HA	DC
ST5	VI(4B)	2 (11.1%)	i11 (13/3/2018)	LTX recipient	ETA	SICU	CO	DC
ST5	VI(4B)		i29 (15/4/2017)	Acute kidney injury	Blood	MSU1	CA	DC
ST239	III(3A)	2 (11.1%)	i13 (14/1/2018)	Urosepsis	Blood	MICU	CA	Death
ST239	III(3A)		i23 (10/6/2018)	Diabetic foot	Tissue	MSU2	CA	DC
ST1535	V(5C2)	3 (16.6%)	i14 (7/1/2018)	Fever with Osteosarcoma	Blood	MSU3	CA	Death
ST1535	V(5C2)		i26 (7/9/2018)	Sepsis	Blood	MSU6	CA	DC
ST1535	V(5C2)		i27 (9/6/2017)	Febrile neutropenia	Blood	BMTU	HA	DC
ST80	IVc(2B)	1 (5.5%)	i15 (10/12/2017)	IAI	Wound	MSU4	CO	DC
ST22	IVa(2B)	1 (5.5%)	i17 (11/10/2017)	Pneumonia in breast cancer	ETA	MICU	HA	death
ST1	V(5C2 and 5),	2 (11.1%)	i18 (9/10/2017)	SSTI	Wound	MSU6	HA	DC
ST1	V(5C2 and 5)		i19 (16/8/2017)	Pneumonia	ETA	MSU6	CA	DC
ST8-like	No SCC detected	1 (5.5%)	i21 (19/3/2018)	Pneumonia	ETA	SICU	CO	Death
ST913	V(5C2)	1 (5.5%)	i32 (15/12/2017)	Diabetic foot	Tissue	MSU2	CA	DC

**BMTU**: bone marrow transplantation unit; **CA**: community-acquired; **CAUTI**: catheter-associated urinary tract infection; **CO**: healthcare-associated colonization; **DC**: hospital discharge; **ETA**: endotracheal aspirate; **HA**: healthcare-associated; **IAI**: intra-abdominal infection; **LTX**: liver transplantation; **MICU**: medical intensive care unit; **MSU**: medical/surgical unit; **SICU**: surgical intensive care unit; **SSI**: surgical site infection; **ST**: sequence type; **SSTI**: skin and soft tissue infection in a drug addict; ***ccr* complex**: cassette chromosome recombinase complex; (*): SCCmec element prediction is based on homology to whole cassette.

**Table 2:** NCBI accession numbers of the 18 sequenced samples.

Sample ID	Specimen	Genome Accession
i01	Blood	JACVZG000000000
i02	ETA	JACYHC000000000
i06	Tissue	JACVZH000000000
i08	Wound	JACVZI000000000
i10	Infected prothesis	JACVZJ000000000
i11	ETA	JACVZK000000000
i13	Blood	JACVZL000000000
i14	Blood	JACVZM000000000
i15	Wound	JACVZN000000000
i17	ETA	JACVZO000000000
i18	Wound	JACYVY000000000
i19	ETA	JACVZP000000000
i21	ETA	JACVZQ000000000
i23	Tissue	JACVZR000000000
i26	Blood	JACYHD000000000
i27	Blood	JACVZS000000000
i29	Blood	JACVZT000000000
i32	Tissue	JACVZU000000000

**Table 3:** Virulome of the sequenced MRSA isolates.

Sequence Type/ SCCmec	Isolate No.	Aureolysin	Serine Proteases	Leucocidins	Enterotoxins	TSST	Exfoliative A	Haemolysins	Staphylokinase	SCIN	Epidermal Cell Diff Inhibitor
ST8-V	i01	*aur*	*splA, splB, splE*	*lukE, lukD*	*seb*	–	–	*hlb, hlgA, hlgB, hlgC*	*sak*	*scn*	–
Stu-VI	i02	–	–	–	–	–	–	–	–	–	–
Stu-VI	i06	*aur*	*splA, splB, splE*	*lukE, lukD*	–	–	–	*hlb, hlgA, hlgB, hlgC*	–	*scn*	–
ST1-like-V	i08	*aur*	*splA, splB, splE*	*lukE*, *lukD, lukS-PV, lukF-PV*	*seq, sek*	–	–	*hlb, hlgA, hlgB, hlgC*	*sak*	*scn*	–
ST121-V	i10	*aur*	*splB*	*lukE, lukD, lukS-PV, lukF-PV*	*seg, seo, seb, sem, sei, seu*	–	–	*hlb, hlgA, hlgB, hlgC*	*sak*	*scn*	–
ST5-VI	i11	–	*splB*	*lukE, lukD*	*seg, seo, seb, sem, sei, seu, ser, sej, sed*	–	–	*hlb, hlgA, hlgB, hlgC*	*sak*	*scn*	–
ST5-VI	i29	*aur*	*splA, splB*	*lukE, lukD*	*seg, seo, seb, sen, sei, seu*	–	–	*hlb, hlgA, hlgB, hlgC*	*sak*	*scn*	–
ST239-III	i13	*aur*	*splA, splB, splE*	*lukE, lukD*	*seq, sek*	–	–	*hlb, hlgA, hlgB, hlgC*	*sak*	–	–
ST239-III	i23	*aur*	*splA, splB, splE*	*lukE, lukD*	*seq, sek*			*hlb, hlgA, hlgB, hlgC*			
ST1535-V	i14	*aur*	*splA, splB, splE*	*lukE, lukD*	–	–	–	*hlb, hlgA, hlgB, hlgC*	–	*scn*	–
ST1535-V	i26	*aur*	*splA, splB, splE*	*lukD*	–	–	–	*hlb, hlgA, hlgB, hlgC*	–	*scn*	–
ST1535-V	i27	*aur*	*splA, splB,, splE*	*lukE, lukD*	–	–	–	*hlb, hlgA, hlgB, hlgC*	–	*scn*	–
ST80-IV	i15	*aur*	*splA, splB*	*lukE, lukD, LukS-PV, lukF-PV*	*sen*	–	–	*hlb, hlgA, hlgB, hlgC*	*sak*	–	*edinB*
ST22-IV	i17	*aur*	–	–	*seo, sei, sen, seg*	*tst*	–	*hlb, hlgA, hlgB, hlgC*		–	–
ST1-V	i18	*aur*	*splA, splB, splE*	*LukE, LukD, LukS-PV, LukF-PV*	*seh, seq, sek*	–	–	*hlb, hlgB*	*sak*	*scn*	–
ST1-V	i19	*aur*	*splA, splB, splE*	*LukE, LukD*	*seh*	–	–	*hlb, hlgC*	*sak*	*scn*	–
ST8-like	i21	*aur*	*splA, splB, splE*	*LukE, LukD*	*seb, sen*	–	–	*hlb, hlgA, hlgB*	*sak*	*scn*	–
ST913-V	i32	*aur*	*splA, splB, splE*	*LukE, LukD*	–	–	*eta*	*hlb, hlgA* *hlgB, hlgC*	*sak*	*scn*	*edinB*

SCIN: *Staphylococcus* Complement Inhibitor; TSST: toxic shock syndrome toxin; (–): no gene was detected.

**Table 4:** Resistance genotypes and phenotypes identified in the MRSA isolates.

Sequence Type/ SCCmec Element	Isolate Serial No.		Resistome and Susceptibility Profile (Genotypic/Phenotypic Data Represented)											
		Fox, β -Lactams ^1^	Penicillin	VA ^3^	CN	E	CIP, LEV	CD	Phenicol ^2^	PSL ^2^	Fuscidic Acid ^2^	DO	MLS	SXT
ST8-V	i01	*mecA*/R	*blaZ*/R	–/S	–/S	–/R	*norA*/S,S	–/R	–	–	–	–/R	–	–/S
STu-VI	i02	*mecA*/R	*blaZ*/R	–/S	*aac(6′)-aph(2″)*/R	–/S	*norA*/S,S	–/S	*Fex A*	–	–	*tet M*/R	–	–/S
STu-VI	i06	*mecA*/R	*blaZ*/R	–/S	*aac(6′)-aph(2″)*, ant *6-Ia, aph(3′)-III*, *aadD*/R	*mph C*/S	–/S,S	*InuA*/S	–	*cfr*	–	–/R	*msrA*	–/S
ST1-like-V	i08	*mecA*/R	*blaZ*/R	–/S	*aac(6′)-aph(2″)*, ant *6-Ia, aph(3′)-III*/R	–/R	*norA*/R,R	–/R	–	–	–	*tet L*/R	–	–/R
ST121-V	i10	*mecA*/R	*blaZ*/R	–/S	*aac(6′)-aph(2″)*/R	–/S	*norA*/R,R	–/S	–	–	–	–/R	–	–/R
ST5-VI	i11	*mecA*/R	*blaZ*/R	–/S	–/S	–/R	*norA*/R,R	–/S	*Fex A*	–	–	*tet M*/R	–	–/R
ST5-VI	i29	*mecA*/R	*blaZ*/R	–/S	–/S	*erm C*/R	*norA*/R,R	–/R	–	–	–	–/R	–	*dfrG*/R
ST239-III	i13	*mecA*/R	*blaZ*/R	–/S	*aac(6′)-aph(2″)*, *aph(3′)-III*/R	*erm C*/R	*norA*/R,R	–/R	*cat (pc221)*	–	–	*tet K*, *tet M*/R	–	–/R
ST239-III	i23	*mecA*/R	*blaZ*/R	–/S	*aac(6′)-aph(2″)*, *aph(3′)-III*/R	*erm C*/R	*norA*/R,R	–/R	–	–	–	*tet K, tet M*/R	–	–
ST1535-V	i14	*mecA*/R	*blaZ*/R	–/S	*aac(6′)-aph(2″), aadD*/R	–/S	*norA*/S,S	–/R	–	–	–	*tet K*/R	–	–/S
ST1535-V	i26	*mecA*/R	*blaZ*/R	–/S	*aac(6′)-aph(2″), aadD*/S	–/S	*norA*/S,S	*InuA*/S	–	–	–	*tet K*/R	–	–/R
ST1535-V	i27	*mecA*/R	*blaZ*/R	–/S	*aac(6′)-aph(2″), aadD*/S	–/S	*norA*/S,S	*InuA*/S	–	–	–	*tet K*/R	–	–/S
ST80-IV	i15	*mecA*/R	*blaZ*/R	–/S	*ant 6-Ia, aph(3′)-III*/S	–/S	*norA*/S,S	–/S	–	–	*fus B*	*tet K*/R	–	–/S
ST22-IV	i17	*mecA*/R	*blaZ*/R	–/S	–/S	*erm C*/S	*norA*/S,S	–/S	–	–	–	*tet L*/R	–	–/S
ST1-V	i18	*mecA*/R	*blaZ*/R	–/S	*aac(6′)-aph(2″), ant 6-Ia, aph(3′)-III*/S	*erm C*/S	*norA*/S,S	–/S	–	–	–	*tet K*/S	–	–/S
ST1-V	i19	*mecA*/R	*blaZ*/R	–/S	*aac(6′)-aph(2″)*/R	–/S	*norA*/R,R	–/S	–	–	–	–/R	–	–/S
ST8-like	i21	*mecA*/R	*blaZ*/R	–/S	–/S	–/S	*norA*/S,S	–/S	–	–	–	–/S	–	–/R
ST913-V	i32	*mecA*/R	*blaZ*/R	–/S	*aac(6′)-aph(2″)*/R	–/S	*norA*/S,S	–/S	–	–	–	–/R	–	–/R

**(–)**: absent gene, **S**: phenotypically sensitive, **R**: phenotypically resistant, **VA**: vancomycin, **CN**: gentamycin, **E**: erythromycin, **CD**: clindamycin, **CIP**: ciprofloxacin, **LEV**: levofloxacin, **SXT**: trimethoprim-sulfamethoxazole, **DO**: doxycycline, **FOX**: cefoxitin. **PSL**: phenicol/streptogramin A/lincosamide multidrug resistance; **^1^** β-lactams tested phenotypically: penicillin, cefoxitin, cefazolin. **^2^** phenicol, PSL and fusidic acid were not tested phenotypically. ^3^ Susceptibility to vancomycin was determined by the E-test.

**Table 5:** Selected statistically significant associations between pairs of variables (a full list in Supplementary Data File 1).

Variable 1	Variable 2	Fisher’s Test *p*-Value	Correlation Coefficient (If Applicable)	Type of Association
*lukS-PV,* *lukF PV*	Severity	0.0237	N/A	Genes absent in severe cases
*aph(3″)-III*	Severity	0.0128	N/A	Gene rare in severe cases, enriched in non-severe ones
*ant.6.Ia*	Severity	0.011	N/A	Gene absent in severe cases
*ant.6.Ia*	Sample	0.003	N/A	Gene enriched in wound isolates
*aur*	*fexA*	0.007	−1	Negative association
*norA*	*msrA, cfr*	>0.05	−1	Negative association
*seg, seo*	*splA*	0.019	−0.679	Negative association
*seg, seo*	*splE*	0.005	−0.756	Negative association
*aur*	*splB*	0.0196	0.79	Positive association
*lukS-PV* *lukF PV*	*ant.6.Ia*	0.019	0.679	Positive association
*lukD*	*splA*	0.0392	0.661	Positive association
*lukD*	*splB*	0.0196	0.791	Positive association
*lukE*	*sak*	0.0245	0.632	Positive association
*aadD*	*sak*	0.005	−0.756	Reverse association

## Supplementary Materials

The following are available online at https://www.mdpi.com/2073-4425/11/10/1219/s1, Figure S1: Two separate correlation matrices (similar to the one in Figure 3) separately indicating all correlations between pairs of virulence genes, as well as pairs of genetic or phenotypic resistance determinants. Shades of blue represent positive correlations, while shades of orange/red represent negative correlations. Table S1: Clinicoepidemiological characteristics of all isolates as well as their deduced sequence types, resistance genes (resistome), virulence genes (virulome) and resistance phenotypes. Table S2: *p*-values for comparisons of all pairs of categorical traits using Fisher’s exact test. The table cells are color-coded for significance with different shades of pink or red. Supplementary Data Sheet 1: Contingency tables and the full results of Fisher’s exact test between pairs of categorical variables which passed the significance threshold (*p*-value < 0.05)
