# Supplementary material for: Genomic Characterization of Methicillin-Resistant Staphylococcus aureus (MRSA) by High-Throughput Sequencing in a Tertiary Care Hospital
Source: Genes (Basel). 2020 Oct 17;11(10):1219. doi: 10.3390/genes11101219 (PMC7603108; doi:10.3390/genes11101219)
Supplement: Supplementary file 1 [file genes-11-01219-s001.zip › genes-936975-supplementary/Supplementary_Data_Sheet1.pdf]

Significant Results ($p < 0.05$) for ALL vs. ALL Fisher's Exact Test Results:

```
aac6.aph2 seb

     0  1
  0  2  4
  1 11  1
Fisher's Exact Test p-value = 0.0217086834733893
------------------------
aac6.aph2 sen

     0  1
  0  2  4
  1 12  0
Fisher's Exact Test p-value = 0.00490196078431372
------------------------
aac6.aph2 CN

    0 1
  0 6 0
  1 3 9
Fisher's Exact Test p-value = 0.0090497737556561
------------------------
aac6.aph2      Sequence.types

    ST1 ST1-like ST121 ST1535 ST22 ST239 ST5 ST8 ST8-like ST80 ST913 STu
  0   0        0     0      0    1     0   2   1        1    1     0   0
  1   2        1     1      3    0     2   0   0        0    0     1   2
Fisher's Exact Test p-value = 0.0144927536231884
------------------------
ant.6.Ia aph3.III

     0  1
  0 12  2
  1  0  4
Fisher's Exact Test p-value = 0.00490196078431373
------------------------
ant.6.Ia lukS.PV

     0  1
  0 13  1
  1  1  3
Fisher's Exact Test p-value = 0.0186274509803922
------------------------
ant.6.Ia lukF.PV

     0  1
  0 13  1
  1  1  3
Fisher's Exact Test p-value = 0.0186274509803922
------------------------
ant.6.Ia Severity

     N  Y
  0  3 11
  1  4  0
Fisher's Exact Test p-value = 0.011437908496732
------------------------
```

ant.6.Ia Sample

	Blood	ETA	Infected prothesis	Tissue	Wound
0	6	5	1	2	0
1	0	0	0	1	3

Fisher's Exact Test p-value = 0.00249875062468766

----

aph3.III sek

	0	1
0	12	0
1	2	4

Fisher's Exact Test p-value = 0.00490196078431372

----

aph3.III seq

	0	1
0	12	0
1	2	4

Fisher's Exact Test p-value = 0.00490196078431372

----

aph3.III SCCmec.element

	I(1B), VI(4B)	III(3A)	IVa(2B)	IVc(2B)	No SCC detected	V(5C2 and 5)	V(5C2)	VI(4B)
0	1	0	1	0	1	1	6	2
1	0	2	0	1	0	2	0	1

Fisher's Exact Test p-value = 0.0359820089955022

----

aph3.III Severity

	N	Y
0	2	10
1	5	1

Fisher's Exact Test p-value = 0.0128205128205128

----

aph3.III Sample

	Blood	ETA	Infected prothesis	Tissue	Wound
0	5	5	1	1	0
1	1	0	0	2	3

Fisher's Exact Test p-value = 0.0199900049975013

----

aadD inuA

	0	1
0	14	0
1	1	3

Fisher's Exact Test p-value = 0.00490196078431373

----

aadD sak

	0	1
0	2	12
1	4	0

Fisher's Exact Test p-value = 0.00490196078431373

----

erm.C sek

	0	1
0	12	1
1	2	3

Fisher's Exact Test p-value = 0.0441176470588235

```
erm.C seq

     0  1
  0 12  1
  1  2  3
Fisher's Exact Test p-value = 0.0441176470588235
------------------------
inuA sak

     0  1
  0  3 12
  1  3  0
Fisher's Exact Test p-value = 0.0245098039215686
------------------------
fex.A tet.M

     0  1
  0 14  2
  1  0  2
Fisher's Exact Test p-value = 0.0392156862745098
------------------------
fex.A aur

     0  1
  0  0 16
  1  2  0
Fisher's Exact Test p-value = 0.00653594771241829
------------------------
fex.A splA

     0  1
  0  2 14
  1  2  0
Fisher's Exact Test p-value = 0.0392156862745098
------------------------
fex.A splB

     0  1
  0  1 15
  1  2  0
Fisher's Exact Test p-value = 0.0196078431372549
------------------------
fex.A    HA.CA.CO

    CA CO HA
  0  9  2  5
  1  0  2  0
Fisher's Exact Test p-value = 0.0384807596201899
------------------------
tet.M aur

     0  1
  0  0 14
  1  2  2
Fisher's Exact Test p-value = 0.0392156862745098
------------------------
tet.M          SCCmec.element

    I(1B), VI(4B) III(3A) IVa(2B) IVc(2B) No SCC detected V(5C2 and 5) V(5C2) VI(4B)
  0             0       0       1       1               1            3      6      2
  1             1       2       0       0               0            0      0      1
Fisher's Exact Test p-value = 0.0439780109945027
------------------------
```

```
aur  splA

     0  1
  0  2  0
  1  2 14
Fisher's Exact Test p-value = 0.0392156862745097
------------------------
aur  splB

     0  1
  0  2  0
  1  1 15
Fisher's Exact Test p-value = 0.0196078431372549
------------------------
aur      HA.CA.CO

    CA CO HA
  0  0  2  0
  1  9  2  5
Fisher's Exact Test p-value = 0.0419790104947526
------------------------
splA splB

     0  1
  0  3  1
  1  0 14
Fisher's Exact Test p-value = 0.00490196078431373
------------------------
splA splE

     0  1
  0  4  0
  1  2 12
Fisher's Exact Test p-value = 0.00490196078431373
------------------------
splA lukD

     0  1
  0  2  2
  1  0 14
Fisher's Exact Test p-value = 0.0392156862745098
------------------------
splA seg

     0  1
  0  1  3
  1 13  1
Fisher's Exact Test p-value = 0.0186274509803922
------------------------
splA sei

     0  1
  0  1  3
  1 13  1
Fisher's Exact Test p-value = 0.0186274509803922
------------------------
splA sem

     0  1
  0  2  2
  1 14  0
Fisher's Exact Test p-value = 0.0392156862745098
------------------------
```

splA seo

	0	1
0	1	3
1	13	1

Fisher's Exact Test p-value = 0.0186274509803922

----

splA Sample

	Blood	ETA	Infected prothesis	Tissue	Wound
0	0	3	1	0	0
1	6	2	0	3	3

Fisher's Exact Test p-value = 0.0239880059970015

----

splB splE

	0	1
0	3	0
1	3	12

Fisher's Exact Test p-value = 0.0245098039215686

----

splB lukD

	0	1
0	2	1
1	0	15

Fisher's Exact Test p-value = 0.0196078431372549

----

splB WardType

	BMTU	MICU	MSU	SICU
0	0	2	0	1
1	1	1	11	2

Fisher's Exact Test p-value = 0.0364817591204398

----

splE seg

	0	1
0	2	4
1	12	0

Fisher's Exact Test p-value = 0.00490196078431372

----

splE sei

	0	1
0	2	4
1	12	0

Fisher's Exact Test p-value = 0.00490196078431372

----

splE seo

	0	1
0	2	4
1	12	0

Fisher's Exact Test p-value = 0.00490196078431372

----

splE seu

	0	1
0	3	3
1	12	0

Fisher's Exact Test p-value = 0.0245098039215686

```
lukS.PV lukF.PV

     0  1
  0 14  0
  1  0  4
Fisher's Exact Test p-value = 0.000326797385620915
------------------------
lukS.PV  Severity

     N  Y
  0  3 11
  1  4  0
Fisher's Exact Test p-value = 0.011437908496732
------------------------
lukS.PV Sample

    Blood ETA Infected prothesis Tissue Wound
  0     6   5                  0      3     0
  1     0   0                  1      0     3
Fisher's Exact Test p-value = 0.00149925037481259
------------------------
lukF.PV  Severity

     N  Y
  0  3 11
  1  4  0
Fisher's Exact Test p-value = 0.011437908496732
------------------------
lukF.PV Sample

    Blood ETA Infected prothesis Tissue Wound
  0     6   5                  0      3     0
  1     0   0                  1      0     3
Fisher's Exact Test p-value = 0.00199900049975012
------------------------
lukE lukD

     0  1
  0  2  1
  1  0 15
Fisher's Exact Test p-value = 0.0196078431372549
------------------------
lukE sak

     0  1
  0  3  0
  1  3 12
Fisher's Exact Test p-value = 0.0245098039215686
------------------------
lukD scn

     0  1
  0  2  0
  1  2 13
Fisher's Exact Test p-value = 0.0441176470588235
------------------------
lukD           SCCmec.element

    I(1B), VI(4B) III(3A) IVa(2B) IVc(2B) No SCC detected V(5C2 and 5) V(5C2) VI(4B)
  0             1       0       1       0               0            0      0      0
  1             0       2       0       1               1            3      6      3
Fisher's Exact Test p-value = 0.0494752623688156
------------------------
```

```
seb seg

     0  1
  0 12  1
  1  2  3
Fisher's Exact Test p-value = 0.0441176470588235
------------------------
seb sei

     0  1
  0 12  1
  1  2  3
Fisher's Exact Test p-value = 0.0441176470588235
------------------------
seb seo

     0  1
  0 12  1
  1  2  3
Fisher's Exact Test p-value = 0.0441176470588235
------------------------
seb seu

     0  1
  0 13  0
  1  2  3
Fisher's Exact Test p-value = 0.0122549019607843
------------------------
seb            Sequence.types

    ST1 ST1-like ST121 ST1535 ST22 ST239 ST5 ST8 ST8-like ST80 ST913 STu
  0   2        1     0      3    1     2   0   0        0    1     1   2
  1   0        0     1      0    0     0   2   1        1    0     0   0
Fisher's Exact Test p-value = 0.0329835082458771
------------------------
seb      WardType

    BMTU MICU MSU SICU
  0    1    3   9    0
  1    0    0   2    3
Fisher's Exact Test p-value = 0.0264867566216892
------------------------
seg sei

     0  1
  0 14  0
  1  0  4
Fisher's Exact Test p-value = 0.000326797385620915
------------------------
seg sem

     0  1
  0 14  0
  1  2  2
Fisher's Exact Test p-value = 0.0392156862745098
------------------------
seg seo

     0  1
  0 14  0
  1  0  4
Fisher's Exact Test p-value = 0.000326797385620915
------------------------
```

```
seg seu

     0  1
  0 14  0
  1  1  3
Fisher's Exact Test p-value = 0.00490196078431373
------------------------
seg            Sequence.types

    ST1 ST1-like ST121 ST1535 ST22 ST239 ST5 ST8 ST8-like ST80 ST913 STu
  0   2        1     0      3    0     2   0   1        1    1     1   2
  1   0        0     1      0    1     0   2   0        0    0     0   0
Fisher's Exact Test p-value = 0.0484757621189405
------------------------
seh  hlgA

     0  1
  0  1 15
  1  2  0
Fisher's Exact Test p-value = 0.0196078431372549
------------------------
sei sem

     0  1
  0 14  0
  1  2  2
Fisher's Exact Test p-value = 0.0392156862745098
------------------------
sei seo

     0  1
  0 14  0
  1  0  4
Fisher's Exact Test p-value = 0.000326797385620915
------------------------
sei seu

     0  1
  0 14  0
  1  1  3
Fisher's Exact Test p-value = 0.00490196078431373
------------------------
sei            Sequence.types

    ST1 ST1-like ST121 ST1535 ST22 ST239 ST5 ST8 ST8-like ST80 ST913 STu
  0   2        1     0      3    0     2   0   1        1    1     1   2
  1   0        0     1      0    1     0   2   0        0    0     0   0
Fisher's Exact Test p-value = 0.0409795102448776
------------------------
sek seq

     0  1
  0 14  0
  1  0  4
Fisher's Exact Test p-value = 0.000326797385620915
------------------------
sek            SCCmec.element

    I(1B), VI(4B) III(3A) IVa(2B) IVc(2B) No SCC detected V(5C2 and 5) V(5C2) VI(4B)
  0             1       0       1       1               1            1      6      3
  1             0       2       0       0               0            2      0      0
Fisher's Exact Test p-value = 0.0414792603698151
------------------------
```

```
sem seo

     0  1
  0 14  2
  1  0  2
Fisher's Exact Test p-value = 0.0392156862745098
------------------------
sem seu

     0  1
  0 15  1
  1  0  2
Fisher's Exact Test p-value = 0.0196078431372549
------------------------
sen            SCCmec.element

    I(1B), VI(4B) III(3A) IVa(2B) IVc(2B) No SCC detected V(5C2 and 5) V(5C2) VI(4B)
  0             1       2       0       0               0            3      6      2
  1             0       0       1       1               1            0      0      1
Fisher's Exact Test p-value = 0.0389805097451274
------------------------
seo seu

     0  1
  0 14  0
  1  1  3
Fisher's Exact Test p-value = 0.00490196078431373
------------------------
seo            Sequence.types

    ST1 ST1-like ST121 ST1535 ST22 ST239 ST5 ST8 ST8-like ST80 ST913 STu
  0   2        1     0      3    0     2   0   1       1    1     1   2
  1   0        0     1      0    1     0   2   0       0    0     0   0
Fisher's Exact Test p-value = 0.0434782608695652
------------------------
seq            SCCmec.element

    I(1B), VI(4B) III(3A) IVa(2B) IVc(2B) No SCC detected V(5C2 and 5) V(5C2) VI(4B)
  0             1       0       1       1               1            1      6      3
  1             0       2       0       0               0            2      0      0
Fisher's Exact Test p-value = 0.0459770114942529
------------------------
seu CIP

     0  1
  0 11  4
  1  0  3
Fisher's Exact Test p-value = 0.0428921568627451
------------------------
seu LEV

     0  1
  0 11  4
  1  0  3
Fisher's Exact Test p-value = 0.0428921568627451
------------------------
hlgA hlgB

     0  1
  0  2  1
  1  0 15
Fisher's Exact Test p-value = 0.0196078431372549
------------------------
```

```
sak CIP

    0 1
  0 6 0
  1 5 7
Fisher's Exact Test p-value = 0.0377073906485671
------------------------
sak LEV

    0 1
  0 6 0
  1 5 7
Fisher's Exact Test p-value = 0.0377073906485671
------------------------
sak            Sequence.types

    ST1 ST1-like ST121 ST1535 ST22 ST239 ST5 ST8 ST8-like ST80 ST913 STu
  0   0        0     0      3    1     0   0   0        0    0     0   2
  1   2        1     1      0    0     2   2   1        1    1     1   0
Fisher's Exact Test p-value = 0.023488255872064
------------------------
scn            SCCmec.element

    I(1B), VI(4B) III(3A) IVa(2B) IVc(2B) No SCC detected V(5C2 and 5) V(5C2) VI(4B)
  0             1       1       1       1               0            0      0      0
  1             0       0       0       0               1            3      6      3
Fisher's Exact Test p-value = 0.00699650174912544
------------------------
scn      Ward.ICU

    BMTU MICU MSU1 MSU2 MSU3 MSU4 MSU5 MSU6 SICU SICU2
  0    0    3    0    0    0    1    0    0    0     0
  1    1    0    2    1    1    0    1    4    2     1
Fisher's Exact Test p-value = 0.0259870064967516
------------------------
scn      WardType

    BMTU MICU MSU SICU
  0    0    3   1    0
  1    1    0   9    3
Fisher's Exact Test p-value = 0.0204897551224388
------------------------
E   CIP

     0  1
  0 10  2
  1  1  5
Fisher's Exact Test p-value = 0.0128205128205128
------------------------
E   LEV

     0  1
  0 10  2
  1  1  5
Fisher's Exact Test p-value = 0.0128205128205128
------------------------
E  CD

     0  1
  0 11  1
  1  1  5
Fisher's Exact Test p-value = 0.00393234216763629
------------------------
```

E Sequence.types

	ST1	ST1-like	ST121	ST1535	ST22	ST239	ST5	ST8	ST8-like	ST80	ST913	STu
0	2	0	1	3	1	0	0	0	1	1	1	2
1	0	1	0	0	0	2	2	1	0	0	0	0

Fisher's Exact Test p-value = 0.0194902548725637

CIP LEV

	0	1
0	11	0
1	0	7

Fisher's Exact Test p-value = 3.14228255404726e-05

CIP SXT

	0	1
0	8	3
1	1	5

Fisher's Exact Test p-value = 0.0497737556561086

LEV SXT

	0	1
0	8	3
1	1	5

Fisher's Exact Test p-value = 0.0497737556561086

Sequence.types SCCmec.element

	I(1B), VI(4B)	III(3A)	IVa(2B)	IVc(2B)	No SCC detected	V(5C2 and 5)	V(5C2)	VI(4B)
ST1	0	0	0	0	0	2	0	0
ST1-like	0	0	0	0	0	1	0	0
ST121	0	0	0	0	0	0	1	0
ST1535	0	0	0	0	0	0	3	0
ST22	0	0	1	0	0	0	0	0
ST239	0	2	0	0	0	0	0	0
ST5	0	0	0	0	0	0	0	2
ST8	0	0	0	0	0	0	1	0
ST8-like	0	0	0	0	1	0	0	0
ST80	0	0	0	1	0	0	0	0
ST913	0	0	0	0	0	0	1	0
STu	1	0	0	0	0	0	0	1

Fisher's Exact Test p-value = 0.000999500249875062

Diagnosis Sample

	Blood	ETA	Infected prothesis	Tissue	Wound
Acute kidney injury	1	0	0	0	0
CAUTI	0	1	0	0	0
Diabetic foot	0	0	0	2	0
Febrile neutropnia	1	0	0	0	0
Fever with osteosarcoma	1	0	0	0	0
IAI	0	0	0	0	1
Infected prosthesis	0	0	1	0	0
LTX recipient	0	1	0	0	0
Osteomyelitis	0	0	0	0	1
Pneumonia	0	3	0	0	0
Sepsis	2	0	0	0	0
SSI	0	0	0	1	0
SSTI	0	0	0	0	1
Urosepsis	1	0	0	0	0

Fisher's Exact Test p-value = 0.00449775112443778

Severity Sample

	Blood	ETA	Infected prothesis	Tissue	Wound
N	0	0	1	3	3
Y	6	5	0	0	0

Fisher's Exact Test p-value = 0.000499750124937531

Ward.ICU WardType

	BMTU	MICU	MSU	SICU
BMTU	1	0	0	0
MICU	0	3	0	0
MSU1	0	0	2	0
MSU2	0	0	2	0
MSU3	0	0	1	0
MSU4	0	0	1	0
MSU5	0	0	1	0
MSU6	0	0	4	0
SICU	0	0	0	2
SICU2	0	0	0	1

Fisher's Exact Test p-value = 0.000499750124937531
