# Supplementary figures and images for: Genomic Characterization of Methicillin-Resistant Staphylococcus aureus (MRSA) by High-Throughput Sequencing in a Tertiary Care Hospital
Source: Genes (Basel). 2020 Oct 17;11(10):1219. doi: 10.3390/genes11101219 (PMC7603108; doi:10.3390/genes11101219)

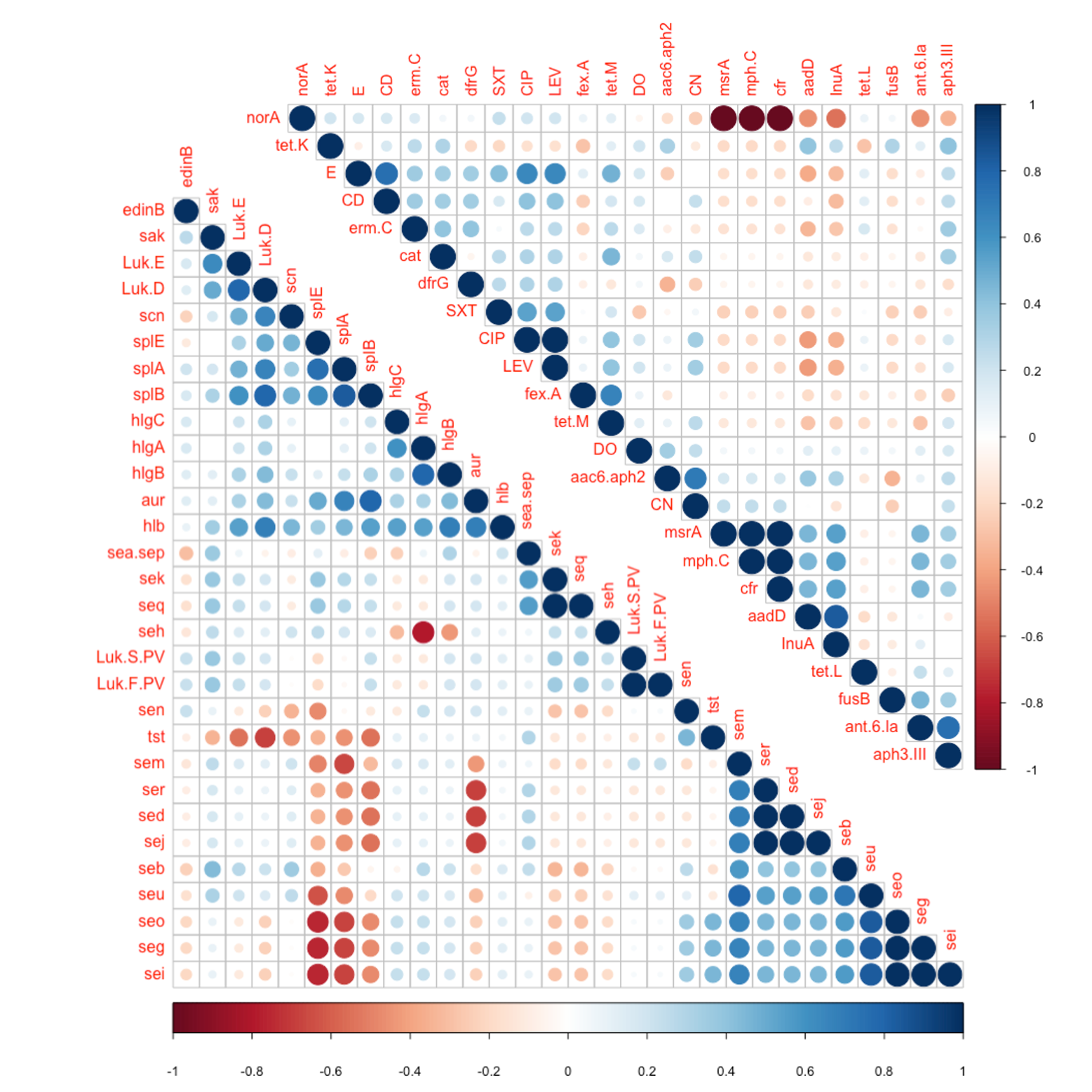

Supplement: Supplementary file 1 [file genes-11-01219-s001.zip › genes-936975-supplementary/FigS1.png]
